# Associations between continuous glucose monitoring (CGM) metrics and psycholinguistic measures: a correlational study

**DOI:** 10.1007/s00592-024-02244-x

**Published:** 2024-03-16

**Authors:** Francesco Marchini, Andrea Caputo, Alessio Convertino, Chiara Giuliani, Olimpia Bitterman, Dario Pitocco, Riccardo Fornengo, Elisabetta Lovati, Elisa Forte, Laura Sciacca, Angela Napoli

**Affiliations:** 1 Centro Italiano Psicologia Analitica (CIPA), Rome, Italy; 2 Department of Clinical, Dynamic and Health Psychology, Sapienza University of Rome, Rome, Italy; 3 Uosd Immunopatologia e allergologia pediatrica, Policlinico Tor Vergata, Rome, Italy; 4 Sapienza University of Rome, Rome, Italy; 5 ASL Roma4, Diabetes Unit, San Paulo Hospital, Civitavecchia, Italy; 6 Diabetes Care Unit Fondazione Policlinico, Universitario Agostino Gemelli IRCCS, Università Cattolica del Sacro Cuore, Rome, Italy; 7 A.S.L. TO4, Chivasso (TO), Piedmont, Italy; 8 Fondazione IRCCS Policlinico San Matteo, Endocrinology, Pavia, Italy; 9 Diabetology Unit, ASL Latina, Latina, Italy; 10 Department of Clinical and Experimental Medicine, Endocrinology Section, University of Catania, Catania, Italy; 11 Israelititco Hospital, International Medical University “Unicamillus” Cdc “Santa Famiglia”, Rome, Italy

**Keywords:** Diabetes, CGM, Mood, Emotions, Depression, Psychological

## Abstract

**Aim:**

Recently, the relationship between diabetes and mental health has been widely studied. With the advent of continuous glucose monitoring (CGM), some researchers have been interested in exploring the association between glucose-related metrics and psychological aspects. These studies have primarily relied on self-report questionnaires which present some limitations.

Therefore, the present multicenter study aims at testing potential associations between CGM metrics and affective processes derived from narratives about using a CGM sensor.

**Methods:**

An exploratory correlational design was used. Fifty-eight adults with type 1 diabetes using CGM were enrolled and invited to complete an online survey, where they replied to an open-ended question regarding their personal experience with the CGM sensor. Texts derived from the answers were analyzed through Linguistic Inquiry and Word Count, a widely used text analysis tool that can automatically identify and quantify linguistic patterns related to various psychological dimensions. Psycholinguistic measures were correlated with CGM metrics.

**Results:**

Higher levels of sadness/depression correlated with lower %TIR (*r* = − 339; *p* < .01) and higher %TAR (*r* = .342; *p* < .01).

**Conclusions:**

The study highlights the relationship between CGM metrics and psychological variables derived from patients' narratives. In particular, it is possible to hypothesize a positive role of %TIR in reducing depressive feelings in individuals with diabetes, as well as a negative role of depressive feelings in achieving desirable CGM outcomes. Additionally, there is a potential role of glycemic variability, particularly hyperglycemia, in the expression of depressive and sad feelings, which has been less studied compared to the effects of hypoglycemia.

## Introduction

In recent years, continuous glucose monitoring (CGM) has emerged as a vital tool in the management of diabetes. CGM systems provide real-time data on glucose levels, enabling individuals with diabetes to make more informed decisions about their treatment and lifestyle choices. Traditional glucose monitoring methods, such as self-monitoring of blood glucose (SMBG), offer limited insight into glucose behavior throughout the day, whereas CGM systems offer a more comprehensive picture of glucose control by providing a continuous stream of glucose measurements. Within the realm of CGM, there has been a growing emphasis on evaluating glycemic control using time in range (%TIR), time above range (%TAR), and time below range (%TBR) as three key measurement indicators, respectively, referring to the percentage (%) of CGM readings, average hours and minutes spent in each range per day, or both, depending on circumstances. As highlighted in recent consensus [1], the principal goal is to increase the TIR—a target range of 70–180 mg/dL—for individuals with type 1 diabetes (T1D) while reducing the TBR (< 70 mg/dL). Achieving the goals for both TBR and TIR results in reduced time spent above range and thus in improved glycemic control. In line with the broad recognition of psychological aspects in diabetes adaptation, the more recent literature has highlighted associations between glucose variability (coefficient of variation “CV”), derived from CGM glucose indicators, and emotions [2–4]. In particular, an association between higher glucose levels and negative mood emerged [2, 4, 5], indicating “hyperglycemia” as a risk factor for mood regulation and mental health. Consistently, with the above-mentioned results, %TIR resulted is associated with higher rates of positive daily mood and lower ratings on most negative mood elements [5–7]. Although these results seem to highlight some associations between glucose variability and mental health/emotions, Mujis et al. (2020) [8] concluded that it is yet unclear how %TIR translates into psychological well-being (mood and cognitive function); as well, the associations between mood and glucose variability have not been convincingly shown. Therefore, further analysis of the relationship between mental health aspects and glucose levels is needed since evidence from scientific studies appears to be heterogeneous [6, 7]. In this regard, two critical methodological aspects can be discussed, which may explain conflicting findings in the current research. First, psychological dimensions are mainly inspected through nonspecific measures based on individual constructs that do not take into account the contextual variability. In other words, it is assumed that affective dimensions are widely referred to the inner psychological status, rather than to specific sense-making processes. For instance, positive or negative mood risks being an overly general and inconclusive variable potentially associated with CGM indicators. Instead, the inspection of positive or negative affect about the CGM use can ensure a higher ecological validity when exploring the interrelations with glucose levels. Second, even when diabetes-related psychological measures are used, they are mostly derived from self-reported questionnaires that are impacted by many biases. For instance, responses can be inflated by low levels of awareness about one's inner world or, on the contrary, by a socially desirable tendency in self-presentation, whereas the use of narratives is deemed as a powerful tool to grasp personal and emotional experiences regarding health issues from a psychosomatic approach [9]. Therefore, to overcome such limitations, we assume that, when examining the relationship between glucose levels and mental health, the individual's affective state should be more CGM-specific and inferred by personal narratives. Based on this premise, the present study aims at testing potential associations between CGM metrics and affective processes derived from narratives about using a CGM sensor.

##  Materials and methods

Adults with T1D were enrolled from five different centers specialized in diabetes care in Italian country. The inclusion criteria were: (a) having type 1 diabetes from at least one year, (b) being aged more than 18 years old, and (c) using a CGM sensor (≥ 70% of time, estimated by the proportion of CGM data downloaded in relation to follow-up time). All participants are using a Dexcom G6 CGM system (Dexcom G6, Dexcom, San Diego, CA, USA). People were recruited and invited to complete an online survey. Informed consent procedures were carried out prior compilation of the survey and required access to CGM data. The survey consisted in two sections: in the first section, people were asked to provide socio-demographic data and diabetes-related information. Then, in the second section, they replied to an open-ended question regarding their personal experience with the CGM sensor use inspired by the written emotional disclosure technique method [9]. Specifically, people were asked to write for ten minutes by letting go and exploring their deepest thoughts and feelings about their experience with the glucose monitoring sensor and the effects it was having on their life, without worrying about grammar, spelling, or sentence structure.^1^ The present study received the ethical approval statement from Ethical Committee of Department of Dynamic, Clinical, and Health Psychology (“Sapienza” University of Rome) and was conducted  according to the Declaration of Helsinki.

### Linguistic inquiry and word count (LIWC)

Texts derived from the answers to the open-ended question were analyzed through Linguistic Inquiry Word Count (LIWC) software [9, 10]. LIWC is an automated text analysis tool, widely used in psychosocial sciences. LIWC examines the degree to which people use different categories of words in a wide range of texts, including emails, speeches, poems, transcripts of daily speech, and open-ended questions. Specifically, the instrument calculates the percentage of words that correspond to various psychological dimensions, such as positive or negative emotions, self-references, causal words, up to 80 psychological dimensions. In particular, this software computes and groups the instances of about 4500 keywords. The dimensions taken into account in this study concern the area of Affective Processes, which includes positive emotions, positive feelings, optimism and energy, negative emotions, anxiety/fear, anger, and sadness/depression.

### Glucose metrics

Glucose CGM metrics were collected within the specialized diabetes centers. For the purpose of the present study, we considered %TIR (percentage of daily values in the standard target range, 70–180 mg/dL.10), %TAR (> 180 mg/dL), %TBR (< 70 mg/dL), and CV (≤ 36%) to represent glucose variability.

### Statistical analysis

Data analysis was performed using IBM SPSS Statistics for Macintosh, Version 24.0. In line with the aim of the present study, correlational analyses were performed (Pearson’s r) between %TIR, %TAR, %TBR, %CV, and psycholinguistic indicators from LIWC related to Affective Processes (emotions positive, negative emotions). In order to analyze the words derived from the collected texts, the Italian LIWC Dictionary (2007) was used.

## Results

### Clinical characteristics of the sample

Sample descriptives are shown in Table 1. Of the 113 adults with T1D who met eligibility criteria, 85 completed the informed consent and the online survey (75.2%). For *27* participants, CGM data over the subsequent 14-day study period were not complete. As a result, the final sample for analysis was composed of 58 participants (51.32%). The majority was female (67.2%), married or living with a partner (74.1%) (educational level, job) and used insulin pump (79.3%). The mean age was 45.88 years (ranging from 18 to 67) and diabetes duration equal to 26.28 years on average. Overall, glycemic control appeared to be in line with the guidance on targets for the assessment of glycemic control for adults with type 1 diabetes [1] since %TIR was 69.58 (± 12.68), CV was 34.67 (± 5.59), and %TBR < 4%.

**Table 1:** Sample descriptives (*N* = 58)

*N* = 58	Mean (SD)
Age (years)	45.88 (12.86)
Female, *n* (%)	39 (67.2%)
Duration of T1D diagnosis (years)	26.28 (± 13.93)
Married or living with a partner, *n* (%)	45 (74.1%)
Insulin pump user, *n* (%)	46 (79.3%)
*CGM metrics*	
Glucose mean (mg/dL)	151.24 (21.64)
% Time in range, 70–180 mg/dL	69.58 (12.68)
% Time below range < 70 mg/dL	3.57 (3.68)
% Time above range > 180 mg/dL	27.00 (13.94)
Coefficient of variation (CV)	34.67 (5.59)
*Psycholinguistic measures (LIWC)*	
Word count	136.57 (140.16)
Affective processes (words—e.g., happy, bad)	6.26 (6.75)
Positive feelings (e.g., happy, joy, love)	4.44 (6.68)
Positive emotions (e.g., happy, good)	1.05 (2.48)
Optimism and energy (e.g., security, pride, victory)	1.35 (1.35)
Negative emotions (e.g., hate, enemy)	1.83 (1.79)
Anxiety or fear (e.g., nervous, fear, tension)	0.64 (0.88)
Anger (e.g., hate, kill)	0.39 (1.22)
Sadness or depression (e.g., to cry, sad)	0.33 (0.65)

### Associations between CGM metrics and psycholinguistic measures

Table 2 shows medium-sized associations of sadness/depression with some CGM metrics. In detail, higher levels of sadness/depression correlated with lower %TIR and higher %TAR. Instead, no further statistically significant associations emerged between CGM metrics and psycholinguistic measures.

**Table 2:** Pearson’s correlations between CGM metrics and psycholinguistic measures

Psycholinguistic measures (LIWC)	%TIR	%TAR	%TBR	%CV
Affective processes (words—e.g., happy, bad)	−0.115	0.065	0.121	0.045
Positive feelings (e.g., happy, joy, love)	− 0.098	0.049	0.136	0.041
Positive emotions (e.g., happy, good)	− 0.209	0.225	− 0.156	− 0.092
Optimism and energy (e.g., security, pride, victory)	− 0.013	− 0.008	0.012	0.141
Negative emotions (e.g., hate, enemy)	− 0.106	0.107	− 0.090	0.007
Anxiety or fear (e.g., nervous, fear, tension)	0.011	− 0.010	− 0.045	0.034
Anger (e.g., hate, kill)	0.016	0.018	− 0.148	− 0.103
Sadness or depression (e.g., to cry, sad)	− 0.339*	0.342*	− 0.151	− 0.061

^*^Correlation is statistically significant at 0.01 level (two-tailed)

## Discussion

This study provides some interesting evidence of the associations between CGM indicators and affective processes derived from patients' narratives. In particular, sadness/depression resulted was negatively associated with %TIR and positively associated with %TAR. These results are in line with the recent literature highlighting the association between %TIR and lower ratings on depression [5] as well as between higher glucose levels and depression [2, 4, 5, 10, 11], since hyperglycemia can lead to higher tiredness/lethargy and cognitive impairments due to decreased energetic arousal [13, 14]. In this regard, it is possible to hypothesize a bidirectional relationship between depressive mood and glycemic control. As highlighted by other studies, individuals who properly utilize the sensor and achieve good levels of glycemic control experience greater satisfaction in terms of quality of life and thus have lower levels of depression [5, 15]. Similarly, the literature has widely concluded that typical feelings associated with depression can hinder self-care processes and adaptation to diabetes, which in turn lead to worse glycemic outcomes [16, 17]. However, it should be noted that our findings only partially confirm the research evidence about the relationship between CGM metrics and negative mood. Indeed, whereas previous studies highlighted the associations of glycemic variability with further negative emotions, such as anger and anxiety [12–14], we found sadness/depression as the unique related variable. Consistently, with what proposed by Marchini et al. (2018) [16] about the connection between unconscious loss processing and overall diabetes adaptation, depressive feelings reveal relevant difficulties in mourning the illness experience. In other words, patients with diabetes reporting depressive feelings about CGM sensor use may be those who experience a sense of self-defectiveness to a higher extent, without hope for reparation. Instead, anxiety and anger feelings may indicate an increased tense arousal in order to defend the self from disruption and activate a process of coping with diabetes challenges and the potential burden of living with CGM (for a review, see Messer et al., 2017 [18]). In this perspective, despite anxiety and anger being negative emotions, they may play an active role and prevent the self from experiencing depressive feelings and a consequent deep narcissistic wound. As well, no evidence emerges about the role of positive emotions in CGM metrics, despite worse glycemic control having been reported as associated with lower feelings of happiness and decreased hedonic tone [13, 14]. This means that positive mood related to CGM sensor use does not necessarily represent a protective factor in glycemic control, probably because it might translate into relying on an external source of regulation without implementing autonomous self-care behaviors [17, 19].

### Conclusions

The study highlights the relationship between metabolic control metrics derived from CGM usage and psychological variables derived from patients' narratives, with specific regard to depressive emotions. While causal links cannot be established between the variables under study, it is possible to hypothesize a positive role of %TIR in reducing depressive feelings in individuals with diabetes, as well as a negative role of depressive feelings in achieving desirable outcomes in the treatment of chronic conditions through new technologies. Additionally, there is a potential role of glycemic variability, particularly hyperglycemia, in the expression of depressive and sad feelings, which has been less studied compared to the effects of hypoglycemia. Future studies should clarify whether the physiological aspect influences the patient's mood or if the mood leads to a deterioration in self-care behaviors and thus glycemic control. In conclusion, it is important to highlight some limitations of the present study, including the number of participants, which does not allow any generalization, and the correlational nature of the study, which does not clarify the role of the study variables in terms of causal effects. This notwithstanding, to our knowledge, the current study represents the first attempt to explore some potential associations between CGM metrics and affective processes, which are directly derived from narratives about using a CGM sensor. Despite depressive emotions about CGM sensor use can be affected by a more global depressive condition, the current study sheds lights on the importance of evaluating them as potential relevant factors in effective glycemic control in patients with diabetes.

## Footnotes

1 The instructions are shown as follows: “In your writing, I would like you to really let go and explore your very deepest emotions and thoughts about your experience with Continuous Glucose Monitoring sensor use and its effects on your life. I would like to know what you are thinking about and feeling in your own words. Once you have started writing, please continue without stopping for about ten consecutive minutes, without worrying about grammar, errors or sentence structure”.
